# Application of a novel deep eutectic solvent modified carbon nanotube for pipette-tip micro solid phase extraction of 6-mercaptopurine

**DOI:** 10.1186/s13065-024-01199-y

**Published:** 2024-04-23

**Authors:** Leila Raisi, Sayyed Hossein Hashemi, Ahmad Jamali Keikha, Massoud Kaykhaii

**Affiliations:** 1 Department of Marine Chemistry, Faculty of Marine Science, Chabahar Maritime University, Chabahar, Iran; 2 Department of Mechanical Engineering, Faculty of Marine Engineering, Chabahar Maritime University, Chabahar, Iran; 3 Department of Chemistry, Faculty of Sciences, University of Sistan and Baluchestan, Zahedan, 98135-674 Iran

**Keywords:** Deep eutectic solvent, Modified carbon nanotube, Pipette-tip micro-solid phase extraction, Seawater analysis

## Abstract

**Background:**

6-mercaptopurine (6-MP) is a chemotherapy drug mainly used to treat leukemia. It is a persistent organic pollutant and can remain in the environment for a long period of time. The presence of 6-MP in the environment poses a number of hazards and needs to be assessed to monitor its potential risk to human health and the environment. However, due to its trace amount in complicated matrices, a clean-up and preconcentration step before its determination is compulsory.

**Results:**

As a highly efficient adsorbent for the extrication of 6-mercaptopurine (6-MP), a novel carbon nanotube doped with camphor: decanoic acid deep eutectic solvent was synthesized and applied as a packing material for the pipette-tip micro solid phase extraction sorbent of 6-MP from tap, wastewater and seawater samples before its spectrophotometric determination. Characteristics and structure of this adsorbent was fully investigated. Factors affecting extraction, including type and volume of the eluent, ionic strength and pH of the sample solution, amount of adsorbent, and number of extraction and elution cycles were optimized using one-factor-at-a-time and response surface methodologies. The method was found to be linear in the range of 1 to 1000 µg/L with a limit of detection and quantification of 0.2 and 0.7 µg/L, respectively. Reproducibility as relative standard deviation was better than 4.6%.

**Conclusion:**

Application of deep eutectic solvent modified carbon nanotube indicated suitable microextraction results and good potential for rapid extraction of trace amounts of 6-MP from different aqueous samples. The amount of sample required for the analysis was less than 10 mL and only 1.5 mg of the adsorbent was used. The total analysis time, including extraction was less than 15 min and the adsorbent could be used for at least 10 times, without significantly losing its adsorption ability. Compared to using unmodified usual carbon nanotubes, deep eutectic solvent doped carbon nanotubes showed 19.8% higher extraction ability.

**Supplementary Information:**

The online version contains supplementary material available at 10.1186/s13065-024-01199-y.

## Introduction

6-mercaptopurine (C_5_H_4_N_4_S, 6-MP) is a well-known chemotherapeutic agent, usually employed for the treatment of different types of cancer and inflammatory bowel diseases including ulcerative colitis and Crohn’s disease. 6-MP is a prodrug, which means that it must be converted into an active form by the body in order to be effective. The compound can inhibit purine metabolism and expansion of the tumor cells. 6-MP can be toxic to aquatic life at even low concentrations. It can be entered into the environment through human waste and wastewater treatment plant effluent, and from manufacturing and disposal facilities. 6-MP is toxic to fish and other aquatic organisms. It can cause death, reproductive impairment, and other problems. It is also toxic to terrestrial organisms, such as birds and mammals. 6-MP is a human carcinogen which can also cause birth defects, reproductive impairment, and other health problems in humans; therefore, it is important to monitor and control the levels of 6-MP in the environment. 6-MP can be present in drinking water that has been contaminated with wastewater treatment plant effluent. Seawater can be contaminated with wastewater treatment plant effluent or manure from livestock that have been treated with 6-MP [1–4]. Monitoring of water can help to track levels of 6-MP in and identify areas where contamination is a problem. This can help to protect drinking water supplies from contamination. And to protect aquatic life from the harmful effects of this chemical.

Because of the complicated and trace amount of the analyte in the real sample, its direct determination is almost impossible. Thus, an extraction step before its instrumental analysis is necessary. In this regard, several adsorbents for the extraction of 6-MP were employed; such as, extraction with molecularly imprinted polymer reinforced with ZnO graphene capped quantum dots [1], metal–organic frameworks and quantum dots [4], modified silver nanoparticles [5], and pencil graphite modified by polypyrrole/functionalized multiwalled carbon nanotubes [6]. The most common instrumentation for detection of 6-MP are high-performance liquid chromatography (HPLC) [7], Raman spectrometry [8], cyclic voltammetry and differential pulse voltammetry [1], electrochemical impedance spectroscopy [1], and spectrophotometry [9].

Solid phase extraction (SPE) is one of the most common extraction techniques which is applied for the analytes enrichment. Recently, miniaturization has become an important trend in SPE development. In this regard, pipette tip micro solid phase extraction (PT-µSPE), as one of the miniaturized formats of SPE, has attracted wide attention [10]. To make a micro SPE column, simply two ordinary pipette tips are connected together. It has advantages such as need for tiny amounts of adsorbent and sample, low solvent consumption, ease of operation, cheapness, and time efficiency [11]. Since PT-µSPE is based on equilibrium rather that exhaustive extraction, its main drawback can be considered as the need for having a highly selective adsorbent, because the number of available adsorbing sites on the solid is limited. As a result, a wide range of adsorbents were employed for PT-µSPE to selectively extract different analytes from a variety of samples, including molecularly imprinted polymers, carbon nanotubes (CNTs), C_18_, C_8_ and silica. Still a lot of attention is focused on the development of new sorbent of this technique to enhance its selectivity, speed, greenness, and so on [12–15].

Due to their high adsorption capacity and rapid desorption, CNTs became attractive for a variety of analytical applications, especially for micro-scale gas and liquid-phase analysis. Moreover, the ease of surface functionalization made CNTs very flexible for the extraction of almost any analyte of interest [16, 17].

Deep eutectic solvents (DESs) are a new class of solvents that can be prepared simply by mixing the appropriate proportions of an acceptor and a donor hydrogen bond compound. DESs represent a promising alternative to conventional organic solvents. They have distinctive features such as a low toxicity and cost of preparation, low flammability, biodegradability, and can be prepared with a wide range of polarities, which make them suitable for any extraction processes, including drugs [18]. For preconcentration of some of drugs, CNTs and poly (methylene blue) redox polymer prepared in ethaline DES [19], DES loaded onto graphene oxide [20], and DES stir bar sorptive extraction [21] were already used which showed excellent results. However, no report we could find on using a CNT coated with a hydrophobic DES for drug extraction.

In this research, a novel adsorbent was prepared for the selective PT-µSPE of 6-MP from aqueous media is presented. Adsorbent was synthesized by modifying CNTs with a DES made of camphor: decanoic acid and fully characterized.

## Experimental

### Apparatus

Spectrophotometric measurements were performed at 210 nm utilizing a Steroglass model Matricola 2016 spectrophotometer (Italy), equipped with 300 µL quartz microcells (model q-01701, Starna, UK). pH of the solutions were measured by a 630 Metrohm pH meter (Switzerland). To investigate spectral interpretation and structural elucidation, a Fourier transform infrared (FTIR) instrument (Perkin-Elmer, Bucks, UK) was employed. A field emission scanning electron microscope (FESEM) (Sigma VP, Zeiss, Germany) was used for taking electron microscope images.

### Reagents

Solid 6-MP (98% purity) was purchased from Sigma-Aldrich (St Louis, MO, USA). All other regents were of analytical grade and obtained from Merck (Darmstadt, Germany). Deionized (DI) water was used for all solution making. A stock of standard solution of 1000 mg/L of 6-MP was obtained by dissolving exactly 102 mg of 6-MP in 20 mL of 0.020 mol/L NaOH and then diluting to 100 mL with DI. This solution was stored at 4 °C in a dark place. Daily standard solutions were acquired by proper diluting of the stock standard solution.

### Preparation of DES-CNT

Three DESs, camphor:decanoic acid, carvone:thymol and camphor:thymol were prepared, each in different ratios of their components. Molar ratios of 1:1, 1:2 and 2:1 were mixed and applied to investigate the performance of DES on the extraction of 6-MP (Additional file 1: Fig. S1). 1.5 mL of each of nine DESs were used for the batch mode extraction of 6-MP from a 10 mL standard solution of 0.380 mg/L for 20 min. Experiments proved better performance of camphor:decanoic acid (1:1). As a result, this DES was selected as the extraction solvent and mixed with CNT. DES-CNT was prepared based on Xu et al. [22] method with small changes. 0.40 g of CNT was poured in methanol. Next, by agitation, various volumes of DES (0.4–2.0 mL) were added and the mixture was sonicated for 1 h. Finally, the sediment DES-CNT was separated by centrifugation at 8000 rpm for 10 min and washed with methanol and dried. These DES-CNTs were used for batch mode extraction of 6-MP from a 10 mL standard solution of 0.380 mg/L for 20 min. As can be seen in Additional file 1: Fig. S2, the highest response was achieved when 1.6 mL volume of DES was mixed with CNT. Consequently, for the experiments below, a DES-CNT composed of 1.6 mL DES camphor:decanoic acid (1:1) with 0.40 g of CNT was applied as extracting medium.

Interactions between the DES, CNT, and 6-MP are shown in Additional file 1: Fig. S3. As depicted, hydrophobic, non-polar interactions of DES can bind it to CNT and also adsorption of 6-MP on DES is due to non-polar interactions.

### PT-µSPE procedure

**Fig. 1:**
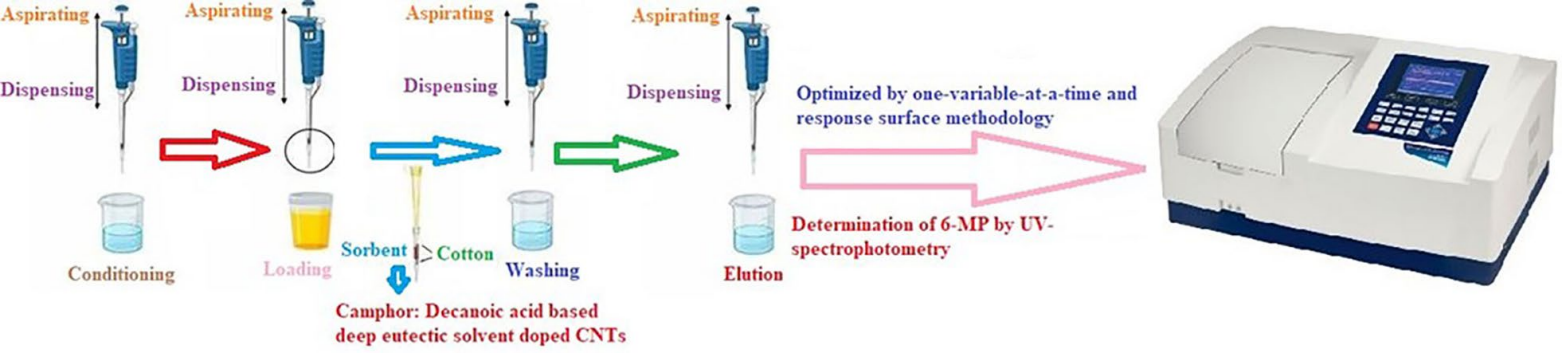
Map of the PT-µSPE device

First a mini column was fabricated by means of two pipette tips of 200 µL and 1000 µL, similar to the work reported by Kaykhaii et al. [10]. 1.5 mg of DES-CNT was firmly immobilized between two pieces of degreased cotton inside the 200 µL pipette tip. Next, a glass syringe was connected to the 1000 µL PT for suction of the sample solution. Next, a glass syringe was connected to the 1000 µL PT for suction of the sample solution. pH of the 10.0 mL of solutions were adjusted to 8.0 by addition of dropwise of 0.1 M NaOH and then 300 mg of NaCl was dissolved in it. To carry out extraction, 500 μL of sample was loaded to the mini column and dispensed back into the sample jar. This operation was repeated ten times. Then, the analyte was eluted by seven passages of 150 μL of acetone: acetic acid (1:9, v/v). Finally, the eluent was transferred into the micro cuvette of the spectrophotometer for determination of 6-MP concentration at a wavelength of 210 nm (Fig. 1).

### Preparation of real sample

Seawater samples were taken from two stations of Chabahar Bay, located at the south east of Iran (Oman Sea). The only pretreatment on the tap water and seawater samples which was performed was their centrifugation at 5000 rpm for 10 min to remove any suspended particles. Wastewater samples were collected from municipal sewage and one of the students’ dormitories. In order to prevent cartridge clogging during µSPE, wastewater samples were filtered through 11 µm particle size retention glass fibre filters under vacuum and then 0.7 µm pore size glass fibre filters. No other pre-treatment was necessary.

## Results and discussion

### FESEM images and FTIR spectra of CNT and DES-CNT

**Fig. 2:**
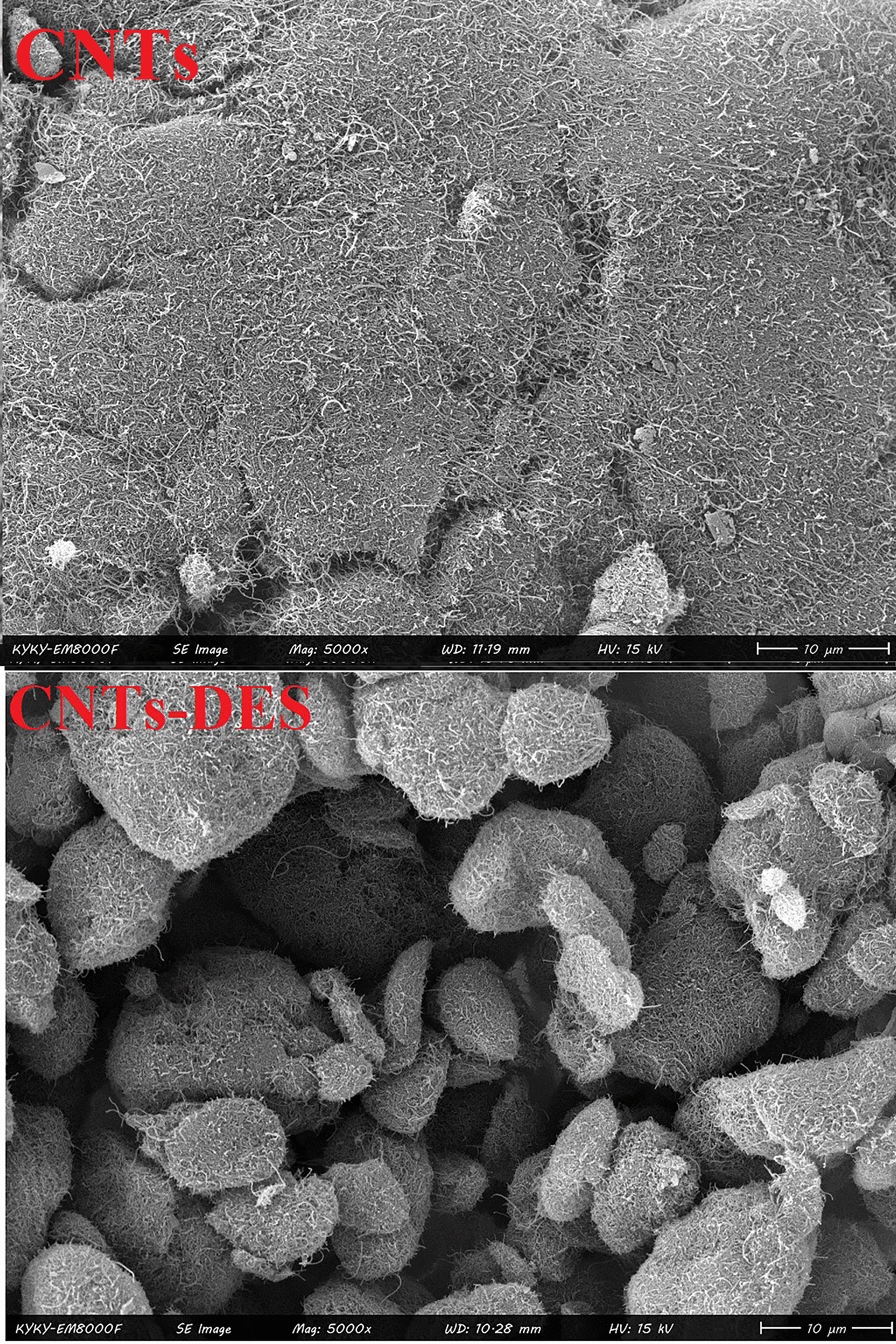
FESEM images of the CNTs and DES-CNTs

**Fig. 3:**
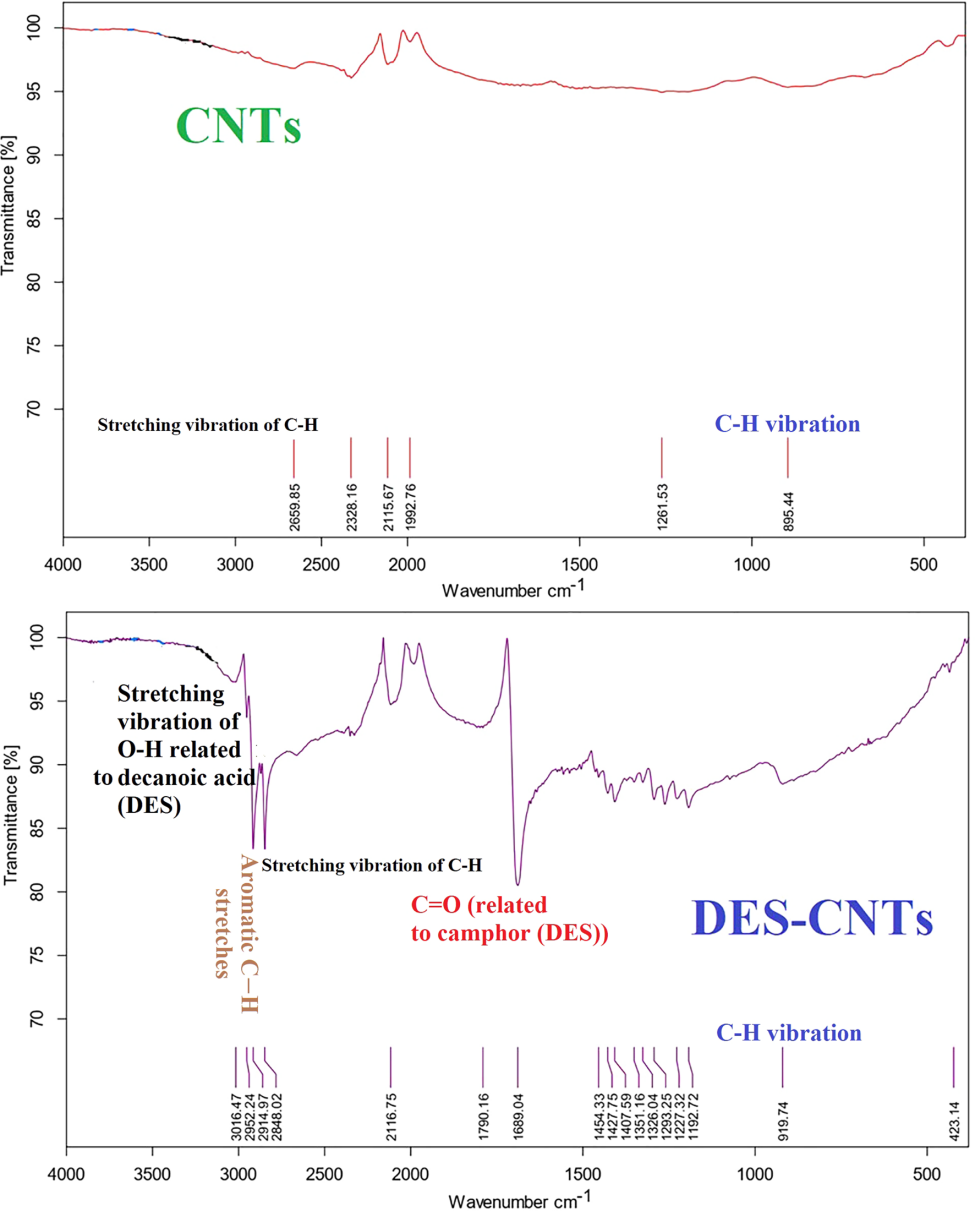
FTIR spectra of CNTs and DES-CNTs

Figure 2 shows FESEM images of the CNT and DES-CNT. As can be seen, they are nanostructured but with two morphologies. Modified CNT has a porous, highly rough, thin tight sheet structure and is wrinkled. It is obvious that the specific surface area of CNT was enhanced by addition of DES which markedly improves the capacity of the sorbent. Figure 3 depicts the FTIR spectra of CNT and DES-CNT. Some additional peaks have appeared in DES-CNT which proves the presence of DES in it.

### Optimization of PT-µSPE

To achieve the highest extraction efficiency of 6-MP, PT-µSPE parameters which could potentially affect the extraction were studied and optimized. Type and volume of the eluent, pH and ionic strength of the solution, the amount of packing material, and the number of extraction and elution cycles were evaluated. The first 4 parameters were optimized by one-factor-at-a-time method and the rest were optimized using response surface methodology (RSM) with signal instrument as the index. A standard aqueous solution of 6-MP with a concentration of 400 µg/L was used for optimization.

#### Effect of the amount of DES-CNT adsorbent

In order to find out how much adsorbent should be placed in the PT, masses between 0.5 to 2.0 mg of DES-CNT was examined. Extraction efficiency (EE) was improved by increasing of mass of adsorbent up to 1.5 mg and then decreased (Additional file 1: Fig. S4). The reason can be due to the formation of a back pressure in higher masses of the adsorbent which led to hard sucking the sample and improper contact of the sample phase with the extracting phase. It was found that 1.5 mg of CNT composite sorbent shows the best EE.

#### Influence of type and volume of the elution solvent

After uptaking the analyte from the sample by the DES-CNT, it should be desorbed from the adsorbent into the minimum solvent (eluent) to have the highest preconcentration factor. Eluent should be able to elute ideally all of the analyte. As a result, the polarity and the volume of the eluting solvent is of importance. Several solvents including acetone, acetonitrile, water, methanol, acetic acid, HCl (0.5 and 1.0 mol/L), and ethanol and different mixture of them were examined. The best EE was obtained by using acetone: acetic acid (1:9, v/v) (Additional file 1: Fig. S5). To examine the effect of eluent volume upon the extraction of 6-MP, different volumes of this solvent at the range of 50 to 200 µL were screened. 150 µL of acetone: acetic acid (1:9, v/v) were found to be optimum for the desorption (Additional file 1: Fig. S6). Lower volumes provided inefficient elution and larger volumes decreased the 6-MP concentration because of dilution.

#### Influence of pH

The pH of the standard solution was changed in the range of pH 2.0 to 12.0 to identify the best pH for the extraction of 6-MP. pH was adjusted by addition of either 0.1 M of NaOH or 0.1 M HCl. The observations showed that the extraction improves up to pH 8.0 and then decreases (Additional file 1: Fig. S7). The reduction of absorbance at lower or higher pH is because of the formation of ionic species of 6-MP. Therefore, the pH of the solutions were adjusted to 8.0 prior doing the extraction.

#### Number of extraction and elution cycles

The number of extraction and elution cycles plays an important role in the enrichment of the compounds in PT-µSPE. Aspiration of the sample solution into the pipette tip and its dispensing back into the same sample vial is called an extraction cycle [10]. Our studies showed that at 10 extraction cycles, the extraction efficiency is maximum (Additional file 1: Fig. S8). At higher number of cycles, the extraction decreased, probably due to the returning back of the analyte to the sample solution. Based on the results, the optimal number of extraction cycles was selected 10 for the further experiments. In order to determine the number of elution cycles, it was varied between 1 and 9. The observations showed that the extraction of 6-MP improves up to 7 cycles and then decreases (Additional file 1: Fig. S9) due to back extraction of the analyte to the sorbent. Thus, 7 elution cycles were chosen as the optimum value.

#### Ionic strength of the sample

Salting-out is a technique which is widely used in many extraction processes. By adding an inorganic salt, the ionic strength of a solution is increased which results in less solubility of organic compounds, hence they can enter more easily to the non-polar extracting solvent. Here, three salts NaCl, KCl and Na_2_SO_4_ were examined for increasing the ionic strength of on extraction of 6-MP; among them, NaCl showed the highest EE and selected as the optimal salt (Additional file 1: Fig. S10). Moreover, the best results were obtained when the concentration of NaCl in the solution was 0.5 M (Additional file 1: Fig. S11).

### Method validation

#### Linearity, limit of detection, limit of quantification, enrichment factor and precision

The established PT-µSPE combined by spectrophotometric protocol was studied in terms of linearity, limit of detection (LOD), limit of quantification (LOQ), and precision. Satisfactory linearity (1–1000 µg/L) was achieved with a ten points external calibration curve. The equation of curve can be expressed as A_(absorbance)_ = 2.1953C_(concentration of 6-MP, mg/L)_ + 0.1159 with a regression coefficient (R^2^) of 0.9924. Relative standard deviation (RSD) of slope and intercept of the calibration curve was 2.9 and 4.1% (n = 3), respectively. A comparison between a standard addition calibration for a wastewater sample spiked by 50 µg/L of 6-MP with the external calibration, indicated a difference less than 4%, which indicates that a simple external calibration can be employed. The LOD (3S_b_) and LOQ (10S_b_) were 0.2 µg/L and 0.7 µg/L, respectively (where S_d_ is standard deviation of 10 measurements of blank solution) [23]. Precision was determined by spiking a seawater sample with 10 µg/L of 6-MP. Upon three consecutive replicate analyses, the intraday and inter-day precision was determined as 3.3% and 4.9%, respectively. For three batches of DES-CNT which were prepared independently, a batch to batch RSD was calculated as 4.6%. The enrichment factor (EF) of the method was obtained as 106 folds by dividing the slope of the calibration curve after DES-CNT-PT-µSPE to the slope of the calibration curve without extraction.

#### Selectivity of the method in the presence of interferences

The usual interferences which normally co-exist in real samples with 6-MP are similar drugs such as levofloxacin, ciprofloxacin, nalidixic acid, acetaminophen and mitoxantrane. In order to study the effect of the interferences on the enrichment of 6-MP in the presence of these potential interfering compounds, aliquots of 10 mL of 380 µg/L of 6-MP was spiked with the same concentration of each of the interferences and analyzed with the developed CNT-DES PT-µSPE method. No interferences were observed for the determination of 6-MP in this condition and the existence of these drugs does not affect the preconcentration and separation of the target analyte.

#### Reusability, stability and adsorption capacity of the prepared CNT-DES

To study the stability and reusability of the prepared CNT-DES, spiked solutions of 6-MP in seawater, tap water and wastewater at a concentration of 50 µg/L were prepared and under the optimized conditions were microextracted by the developed method five times, using the same adsorbent. After each experiment, CNT-DES was washed various times by acetone: acetic acid (1:9, v/v) to be sure of no memory effect. A slight decrease in extraction efficiency was obtained after the four adsorption-elution cycles and after five times, the extraction efficiency was dropped by 5.6%. This result shows that the prepared adsorbent has proper stability and reusability.

To calculate the adsorption capacity of the prepared CNT-DES, a 1000 mg/L solution of 6-MP was extracted with 1.5 mg of the adsorbent under the optimum conditions. The adsorption capacity was obtained as 250 µg/g of the sorbent for 6-MP uptake based on the equation q = (C_i_–C_e_)V/m [16], where q is the maximum adsorption capacity (mg/g), C_i_ is the initial concentration of the standard solution (mg/L), C_e_ is the concentration after extraction (962.5 mg/L), V is the volume of the sample solution (10 mL) and m is the mass of adsorbent (g).

#### Selectivity of the procedure in the presence of interferences

The usual interferences which normally co-exist in real samples including 6-MP are drugs such as levofloxacin, ciprofloxacin, nalidixic acid, acetaminophen and mitoxantrane. To study the effect of the interferences on the enrichment of 6-MP, aliquots of 10 mL of 380 µg/L of 6-MP was spiked with the same concentration of each of the interferences and analyzed with the proposed PT-µSPE. No interferences were observed for the determination of 6-MP.

#### Real sample analysis

In order to find applicability of the developed method for the analysis of real samples, aqueous samples with different matrices (i.e. tap water, seawater and wastewater) were subjected to the analysis (Table 1). No analyte was detected in seawater and tap water samples and the concentration of 6-MP in wastewater was 1.9 µg/L. In order to investigate the effect of sample matrix, recovery experiments were conducted by spiking the same samples at three concentration levels of 10, 20, and 50 µg/L of 6-MP. Recoveries were obtained by using the equation: *relative recovery(%)* = *(C*_*found*_ *−* *C*_*sample*_*)/C*_*added*_ × *100*. In which, C_sample_, C_found_ and C_added_ are the amount of 6-MP found in the real sample before spiking, its concentration after spiking, and the concentration of the standard used for the spiking of the sample solution, respectively. Recoveries were in the range of 97.0–99.6% with RSDs better than 4.6%.The obtained performance of DES-CNT-PT-µSPE and data detail of the LOD and LOQ are summarized in Additional file 1: Tables S1 and S2.

**Table 1:** Results of the analysis of real samples

Sample	Analyte added (µg/L)	Analyte found (µg/L)	Recovery (%)	RSD% (n = 3)
Seawater (taken from Tis coast)	0	Not detected	–	–
	10	9.8	98.0	2.1
	20	19.7	98.5	3.6
	50	49.8	99.6	3.1
Seawater (taken from Haft Tir coast, Persian Gulf)	0	Not detected	–	–
	10	9.9	99.0	2.7
	20	19.8	99.0	3.2
	50	49.6	99.2	4.1
Tap water	0	Not detected	–	–
	10	9.9	99.0	2.9
	20	19.4	97.0	3.3
	50	49.0	98.0	3.8
Wastewater	–	1.9	–	3.0
	10	11.6	97.0	1.1
	20	21.3	97.0	2.7
	50	51.3	98.8	4.6

**Fig. 4:**
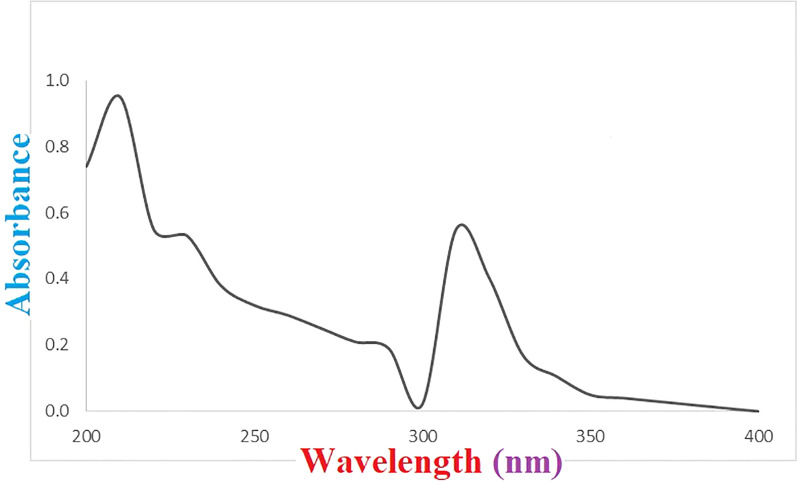
Spectrum of 6-MP against blank solution after DES-CNT-PT-µSPE of a seawater sample (taken from Tis coast) spiked with 380 µg/L of the analyte

It can be concluded that the suggested protocol is a good and simple way for the determination of 6-MP in water and seawater samples. Figure 4 depicts the absorbance spectra of a seawater sample spiked with 380 µg/L of 6-MP which was extracted by the developed DES-CNT-PT-µSPE method. As can be seen, no interferences were observed.

#### Comparison of the performance of the method with similar researches

**Fig. 5:**
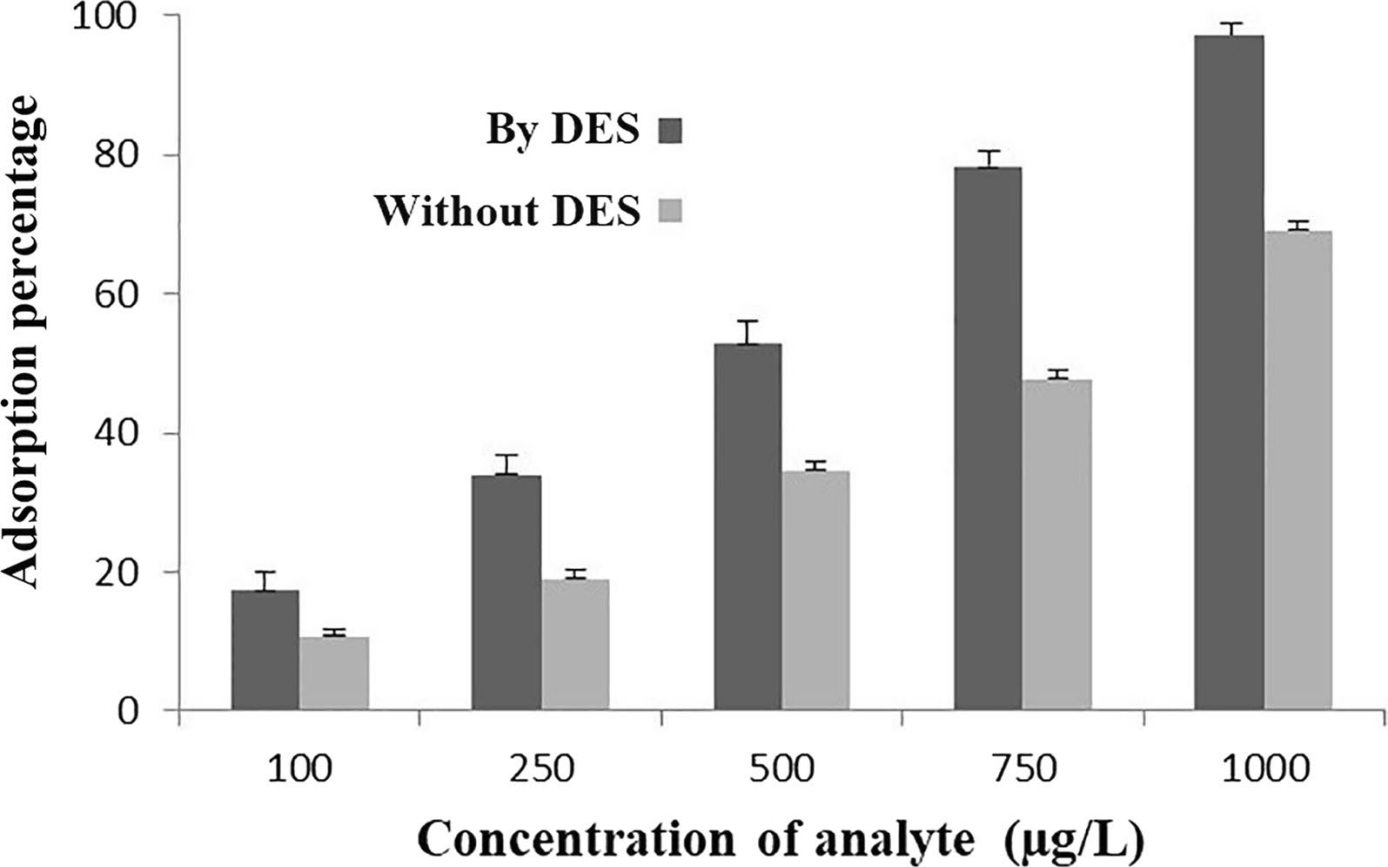
Comparison of CNTs- PT-µSPE and DES-CNT-PT-µSPE

With the aim of finding the effect of doping of DES into CNTs on the microextraction of 6-MP, untreated CNT and DES-CNT were subjected to the extraction of this analyte from five standard solutions with the concentrations of 100, 250, 500, 750 and 1000 µg/L under the same (optimized) condition. As can be seen in Fig. 5 for all standard solutions, in average, extraction by CNT-DES excessed > 19.8% increase in EE compared to non-functionalized CNT.

As mentioned in the Introduction section, different methods of extraction were utilized for the isolation of 6-MP at low levels from various matrices. Table 2 compares the characteristic data of the present method with those reported in literature. As can be seen, the developed method has a lower LOD, higher linear dynamic range and comparable recoveries in compared with the methods recently published in the literature. While electrochemical methods generally have lower LODs than spectrophotometric methods, as shown in the Table, the LOD of the developed method is close to those of cyclic voltammetry and differential pulse voltammetry [1, 24]. This is due to the higher capacity of the DES-CNT used in PT-µSPE. However, the selectivity of those methods is higher because they utilize MIPs, and, due to the nature of electrochemical methods, these detection instruments offer a wider linear range. Despite the application of a fluorescence detector with silver nanoparticles [4] for the extraction of 6-MP, it resulted in a higher LOD with a shorter linear range of the calibration curve. This is likely due to limitations in the capacity of the sorbent. Moreover, DES-CNT exhibits good reusability and stability. Another advantage of this method is the consumption of only 1.5 mg of sorbent, which is significantly less than typical microextraction methods. For example, a pencil graphite electrode modified with polypyrrole/functionalized multi-walled carbon nanotubes combined with differential pulse voltammetry [24] requires 100 mg of multi-walled carbon nanotubes for 6-MP extraction.

**Table 2:** Comparison of suggested DES-CNT-PT-µSPE with recent researches for determination of 6-mercaptopurine

Sample matrix	Preconcentration method	Instrument	Linear range (µg/L)	LOD (µg/L)	Refs.
Pills, human plasma and urine	MIP reinforced by ZnO graphene capped quantum	Cyclic voltammetry, differential pulse voltammetry	0.01–700.00 µM	5.72 µM	[1]
Pharmaceuticals	Silver nanoparticles	Fluorescence spectroscopy	83.70–8370	24.35	[5]
Human urine	Not mentioned	HPLC with fluorescence detector	0.0615–2.40	0.05	[7]
Pharmaceutical samples	Not mentioned	Spectrophotometer	412–2884	12	[9]
Drug	Pencil graphite electrode modified with polypyrrole/functionalized multiwalled carbon nanotubes	Differential pulse voltammetry	30.44–152.18	12.17	[24]
Seawater, wastewater, tap water	DES-CNT-PT-µSPE	Spectrophotometer	1.0–1000.0	0.2	This work

## Conclusion

In this research, a novel camphor: decanoic acid DES doped CNT composite sorbent was successfully prepared and was used for pipette tip micro-SPE of 6-MP from water and wastewater samples. The new sorbent showed excellent adsorption characteristics. Results showed that this sorbent can provide higher response and extraction in comparison to untreated CNT or DES alone. Under optimized conditions, the linear range was between 1 to 1000 µg/L. The whole method could be applied for the determination of 6-MP in an aqueous sample in less than 15 min. The sorbent could be prepared in two easy steps and could be reused at least 10 times. The adsorptive capacity of the DES-CNT was found to be 250 µg/g and reproducibility of the method was ≤ 4.6%. In overall, DES-CNT-PT-µSPE has a good potential for the isolation and preconcentration of 6-MP from complex aqueous matrices.

### Supplementary Information

**Additional file 1: Fig. S1.** Effect of deep eutectic solvent (DES) type on the extraction efficiency. **Fig. S2.** Effect of volume of DES on the extraction efficiency. **Fig. S3.** Interaction of CNT, camphor:decanoic acid DES, and 6-MP. **Fig. S4.** Effect of amount of DES-CNT on the extraction efficiency. **Fig. S5.** Effect of type of eluent on the extraction efficiency. **Fig. S6.** Effect of volume of eluent on the extraction efficiency. **Fig. S7.** Effect of pH on the extraction efficiency. **Fig. S8.** Effect of number of extraction cycles on the extraction efficiency. **Fig. S9.** Effect of number of elution cycles on the extraction efficiency. **Fig. S10.** Effect of type of salt on the extraction efficiency. **Fig. S11.** Effect of amount of NaCl on the extraction efficiency. **Fig. S12.** Response surface-2D/contours including the effect of the independent variable on the extraction efficiency of 6-MP. **Table S1.** The obtained performance of DES-CNT-PT-µSPE. **Table S2.** Data details of the LOD and LOQ. **Table S3.** The design of the actual experiments. **Table S4.** Analysis of variance of BBD model for 6-MP extraction.

## Data Availability

The majority of the data used to support the findings of this study are included within the article. Other data are available from the corresponding author upon request.

## Abbreviations

BBD: Box–Behnken design
CNTs: Carbon nanotubes
CNT-DES-PT-µSPE: Deep eutectic solvent-doped CNTs as an efficient adsorbent for pipette-tip micro-solid phase extraction
DESs: Deep eutectic solvents
DI: Deionized
DES-CNT: DES doped CNT
DES-CNTs: DES doped CNTs
EE: Extraction efficiency
FESEM: Field emission scanning electron microscope
FTIR: Fourier transform infrared spectroscopy
f: Signal function
LOD: Limit of detection
LOQ: Limit of quantification
6-MP: 6-Mercaptopurine
PT-µSPE: Pipette-tip micro Solid-phase extraction
R^2^adj: Coefficient of adjusted R^2^
R^2^: Coefficient of determination
R^2^pred: Coefficient of predicted
R^2^: Regression coefficient
RSD: Relative standard deviation
RSM: Response surface methodology
S_d_: Standard deviation
SPE: Solid-phase extraction
TCPO: Bis-(2, 4, 6-trichlorophenyl) oxalate

## References

[1] Hatamluyi B, Es'haghi Z (2018). Electrochemical biosensing platform based on molecularly imprinted polymer reinforced by ZnO graphene capped quantum dots for 6-mercaptopurine detection. Electrochim Acta.

[2] Sahasranaman S, Howard D, Roy S (2008). Clinical pharmacology and pharmacogenetics of thiopurines. Eur J Clin Pharmacol.

[3] Kanemitsu H, Yamauchi H, Komatsu M, Yamamoto S, Okazaki S, Uchida K, Nakayama H (2009). 6-Mercaptopurine (6-MP) induces cell cycle arrest and apoptosis of neural progenitor cells in the developing fetal rat brain. Neurotoxicol Teratol.

[4] Jin M, Mou ZL, Zhang RL, Liang S, Zhang Z (2017). An efficient ratiometric fluorescence sensor based on metal-organic frameworks and quantum dots for highly selective detection of 6-mercaptopurine. Biosens Bioelectron.

[5] Biparva P, Abedirad SM, Kazemi SY (2015). Silver nanoparticles enhanced a novel TCPO-H_2_O_2_ safranin O chemiluminescence system for determination of 6-mercaptopurine. Spectrochim Acta A.

[6] Karimi-Maleh H, Fallah Shojaei A, Karimi F, Tabatabaeian K, Shakeri S, Moradi R (2016). Simultaneous determination of 6-mercaptopruine, 6-thioguanine and dasatinib as three important anticancer drugs using nanostructure voltammetric sensor employing Pt/MWCNTs and 1-butyl-3-methylimidazolium hexafluoro phosphate. Biosens Bioelectron.

[7] Li AP, Peng JD, Zhou MQ, Zhang J (2016). Resonance light scattering determination of 6-mercaptopurine coupled with HPLC technique. Spectrochim Acta part A.

[8] Han G, Liu R, Han MY, Jiang C, Wang J, Du S, Liu B, Zhang Z (2014). Label free surface-enhanced Raman scattering imaging to monitor the metabolism of antitumor drug 6-mercaptopurine in living cells. Anal Chem.

[9] Tu C, Wen X (2014). Spectrophotometric determination of 6-mercaptopurine in pharmaceutical sample using Fe(III)-potassium ferricyanide system. Adv Mat Res.

[10] Kaykhaii M, Yavari E, Sargazi G, Khajeh EA (2020). Highly sensitive determination of bisphenol A in bottled water samples by HPLC after Its extraction by a novel Th-MOF pipette-tip micro-SPE. J Chromatogr Sci.

[11] Tamandani M, Hashemi SH (2022). Central composite design (CCD) and Box-Behnken design (BBD) for the optimization of a molecularly imprinted polymer (MIP) based pipette tip micro-solid phase extraction (SPE) for the spectrophotometric determination of chlorpyrifos in food and juice. Anal Lett.

[12] Arabi M, Ostovan A, Li J, Wang X, Zhang Z, Choo J, Chen L (2021). Molecular imprinting: green perspectives and strategies, advanced materials. Adv Mater.

[13] Arabi M, Ostovan A, Bagheri AR, Guo X, Wang L, Li J, Wang X, Li B, Chen L (2020). Strategies of molecular imprinting-based solid-phase extraction prior to chromatographic analysis. TrAC - Trends Anal Chem.

[14] Ostovan A, Arabi M, Wang Y, Li J, Li B, Wang X, Chen L (2022). Greenificated molecularly imprinted materials for advanced applications. Adv Mater.

[15] Arabi M, Ostovan A, Bagheri AR, Guo X, Li J, Ma J, Chen L (2020). Hydrophilic molecularly imprinted nanospheres for the extraction of rhodamine B followed by HPLC analysis: A green approach and hazardous waste elimination. Talanta.

[16] Tamandani M, Hashemi SH (2022). Spectrophotometric determination of chlorpyrifos in foodstuff after pipette-tip micro solid extraction by modified carbon nanotube. J Food Compost Anal.

[17] Hashemi SH, Yahyavi H, Kaykhaii M, Hashemi M, Mirmoghaddam M, Keikha AJ (2019). Spectrofluorometrical determination of vitamin B_1_ in different matrices using Box-Behnken designed pipette tip solid phase extraction by a carbon nanotube sorbent. Chem Select.

[18] Chełstowska PM, Kaykhaii M, Wasylka JP, Guardia M (2022). Magnetic deep eutectic solvents—fundamentals and applications. J Mol Liq.

[19] Hosu O, Barsan MM, Săndulescu R, Cristea C, Brett CMA (2021). Hybrid Nanocomposite platform, based on carbon nanotubes and poly(methylene Blue) redox polymer synthesized in ethaline deep eutectic solvent for electrochemical determination of 5-aminosalicylic acid. Sensors (Basel).

[20] Jangizahi J, Hashemi SH (2023). Deep eutectic solvent loaded onto graphene oxide as a novel phase for pipette-tip micro-solid phase extraction of levofloxacin from seawater and body fluid samples. Int J Environ Anal Chem.

[21] Hashemi SH, Kaykhaii M, Jamali Keikha A, Raisi L (2023). Deep eutectic solvent stir bar sorptive extraction: a rapid microextraction technique for the determination of vitamin D_3_ by spectrophotometry. ACS Omega.

[22] Xu K, Wang Y, Zhang HH, Yang Q, Wei X, Xu P, Zhou Y (2017). Solid-phase extraction of DNA by using a composite prepared from multiwalled carbon nanotubes, chitosan, Fe_3_O_4_ and a poly(ethylene glycol)-based deep eutectic solvent. Microchim Acta.

[23] Zhang N, Hu B (2012). Cadmium (II) imprinted 3-mercaptopropyltrimethoxysilane coated stir bar for selective extraction of trace cadmium from environmental water samples followed by inductively coupled plasma mass spectrometry detection. Anal Chim Acta.

[24] Karimi-Maleh H, Tahernejad-Javazmi F, Atar N, Yola MLT, Gupta VK, Ensafi AA (2015). A novel DNA biosensor based on a pencil graphite electrode modified with polypyrrole/functionalized multiwalled carbon nanotubes for determination of 6-mercaptopurine anticancer drug. Ind Eng Chem Res.

