# Supplementary material for: Application of a novel deep eutectic solvent modified carbon nanotube for pipette-tip micro solid phase extraction of 6-mercaptopurine
Source: BMC Chem. 2024 Apr 23;18(1):81. doi: 10.1186/s13065-024-01199-y (PMC11040795; doi:10.1186/s13065-024-01199-y)
Supplement: Supplementary file 1 — Additional file 1: Fig. S1. Effect of deep eutectic solvent (DES) type on the extraction efficiency. Fig. S2. Effect of volume of DES on the extraction efficiency. Fig. S3. Interaction of CNT, camphor:decanoic acid DES, and 6-MP. Fig. S4. Effect of amount of DES-CNT on the extraction efficiency. Fig. S5. Effect of type of eluent on the extraction efficiency. Fig. S6. Effect of volume of eluent on the extraction efficiency. Fig. S7. Effect of pH on the extraction efficiency. Fig. S8. Effect of number of extraction cycles on the extraction efficiency. Fig. S9. Effect of number of elution cycles on the extraction efficiency. Fig. S10. Effect of type of salt on the extraction efficiency. Fig. S11. Effect of amount of NaCl on the extraction efficiency. Fig. S12. Response surface-2D/contours including the effect of the independent variable on the extraction efficiency of 6-MP. Table S1. The obtained performance of DES-CNT-PT-µSPE. Table S2. Data details of the LOD and LOQ. Table S3. The design of the actual experiments. Table S4. Analysis of variance of BBD model for 6-MP extraction. [file 13065_2024_1199_MOESM1_ESM.docx]

**Additional data to:**

**Application of a novel deep eutectic solvent modified carbon nanotube for pipette-tip micro solid phase extraction of 6-mercaptopurine**

*Leila Raisi^1^,* *Sayyed Hossein Hashemi^1^, Ahmad Jamali Keikha^2^ and Massoud Kaykhaii^3,^^[[1]](#footnote-1)^*

**
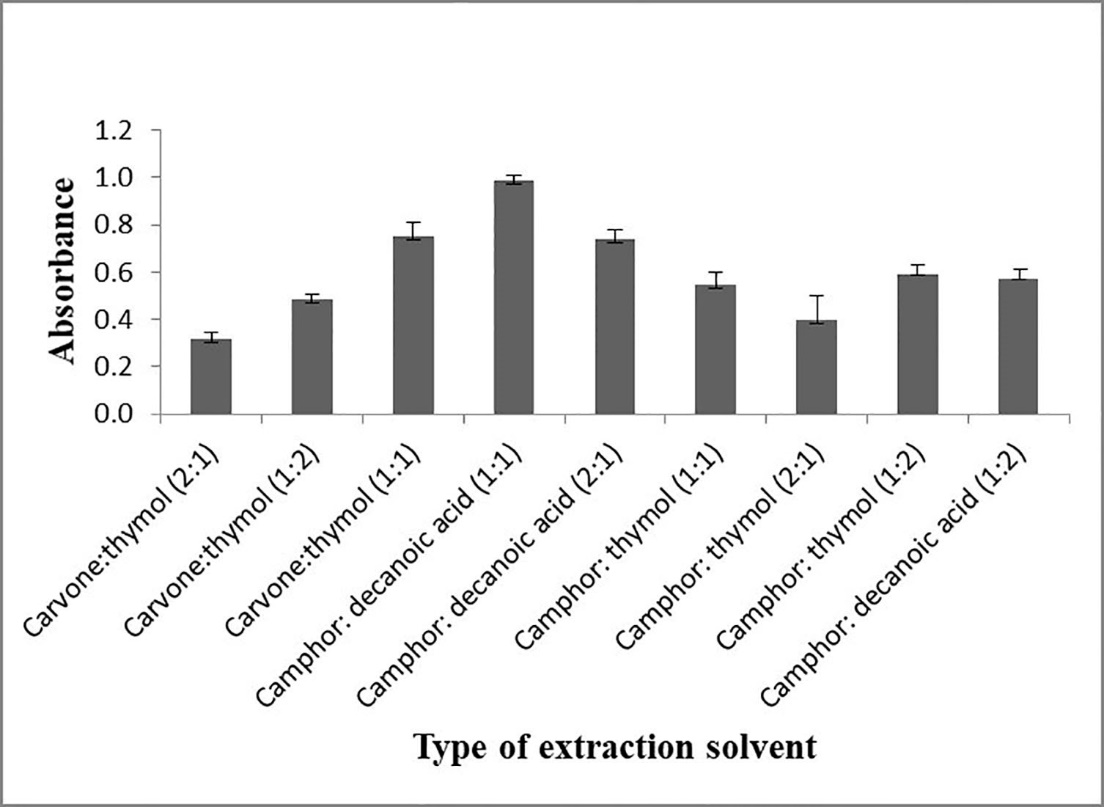
**

**Fig. S1.** Effect of deep eutectic solvent (DES) type on the extraction efficiency.

**
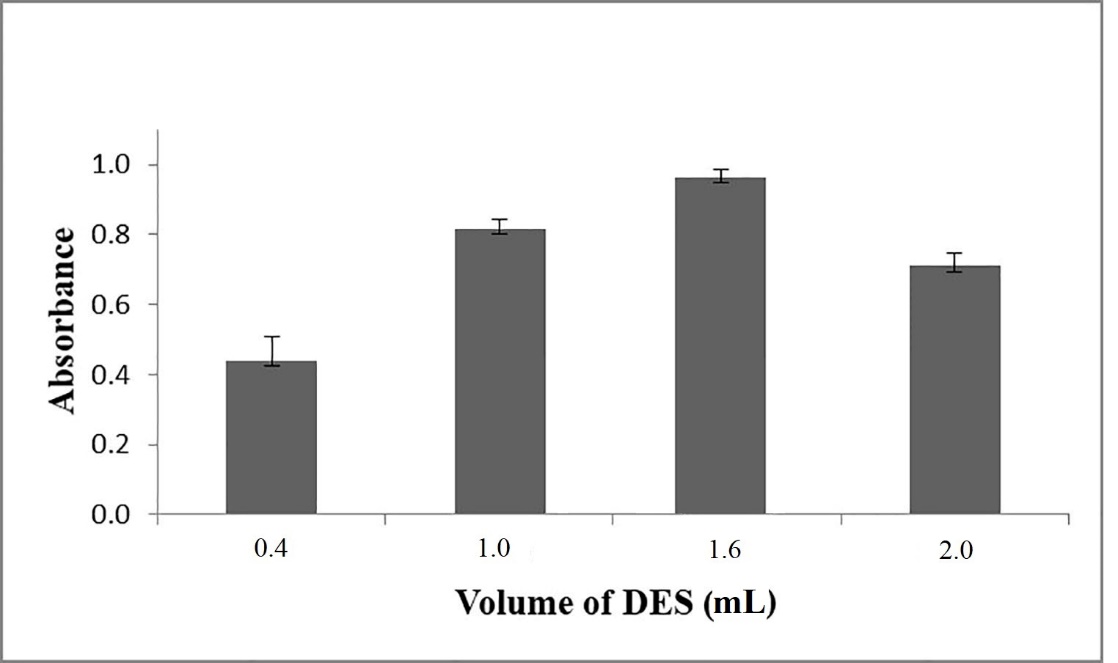
**

**Fig. S2.** Effect of volume of DES on the extraction efficiency.

**
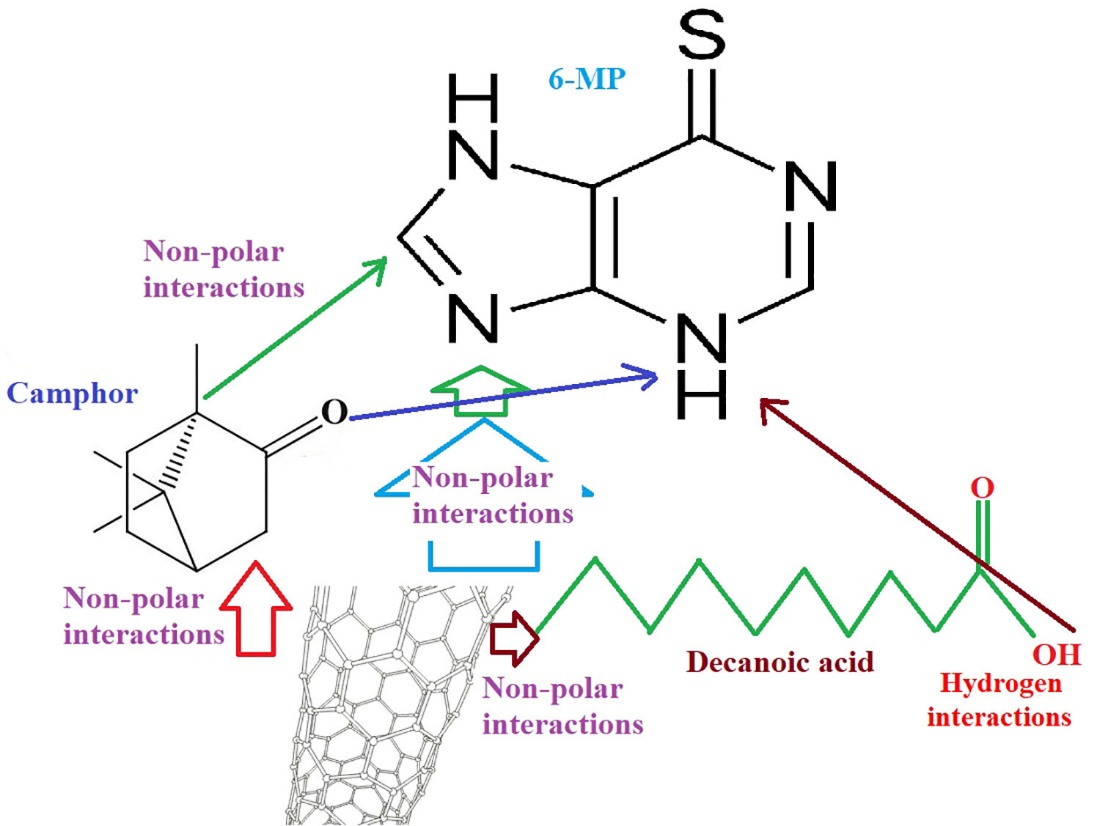
**

**Fig. S3.** Interaction of CNT, camphor:decanoic acid DES, and 6-MP.

***
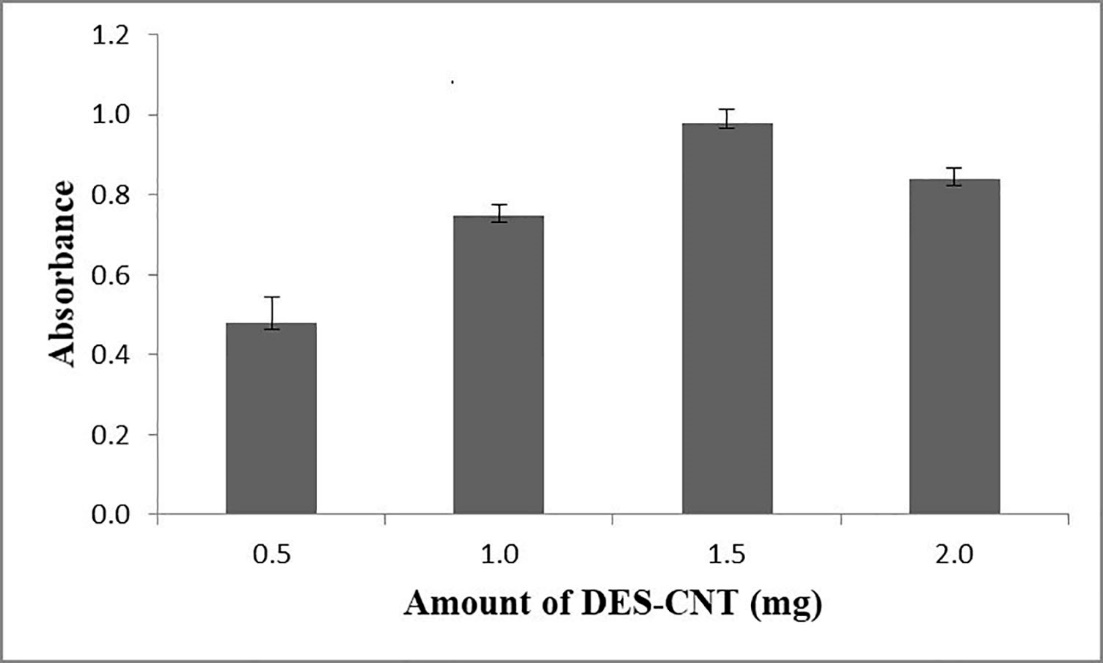
***

**Fig. S4.** Effect of amount of DES-CNT on the extraction efficiency.

**
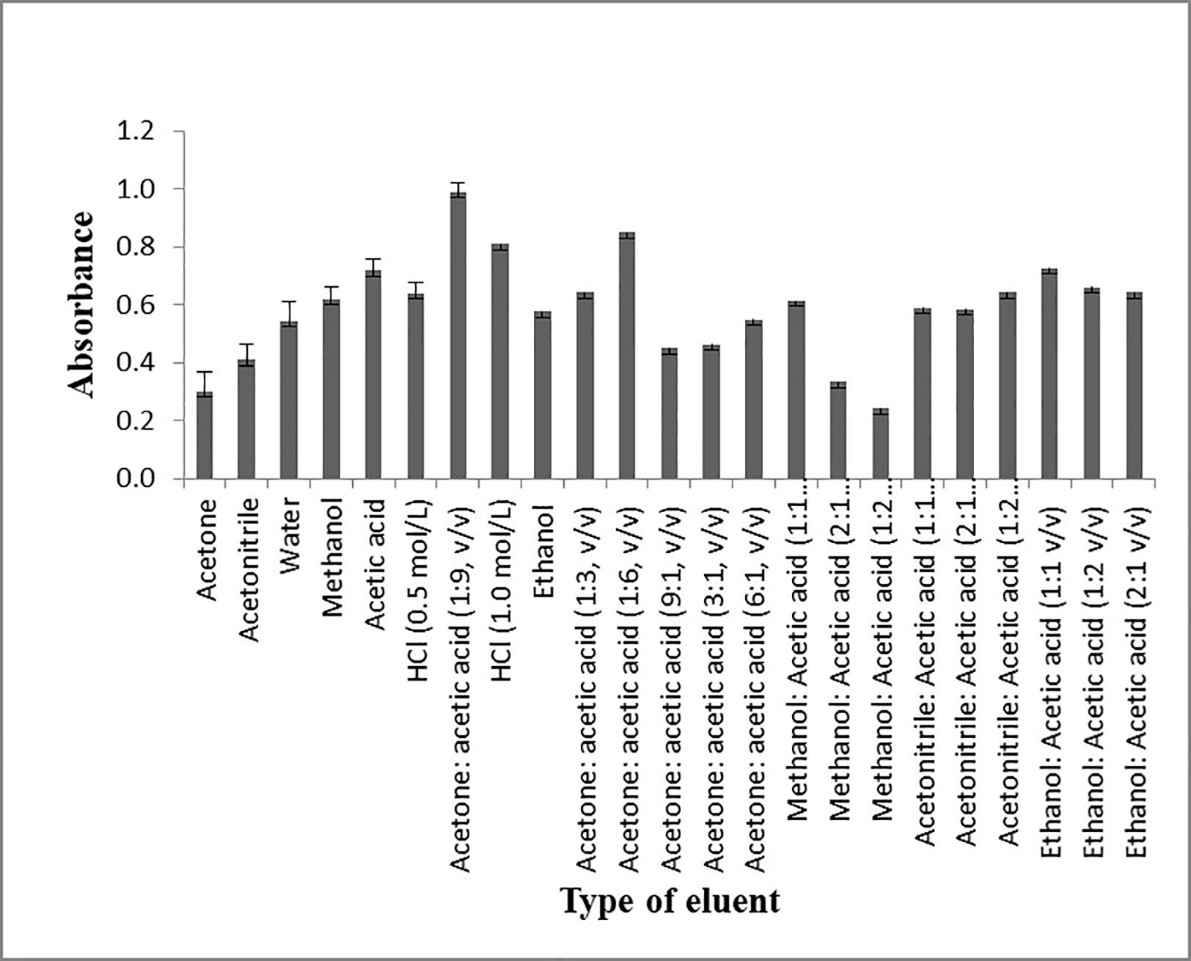
**

**Fig. S5.** Effect of type of eluent on the extraction efficiency.

**
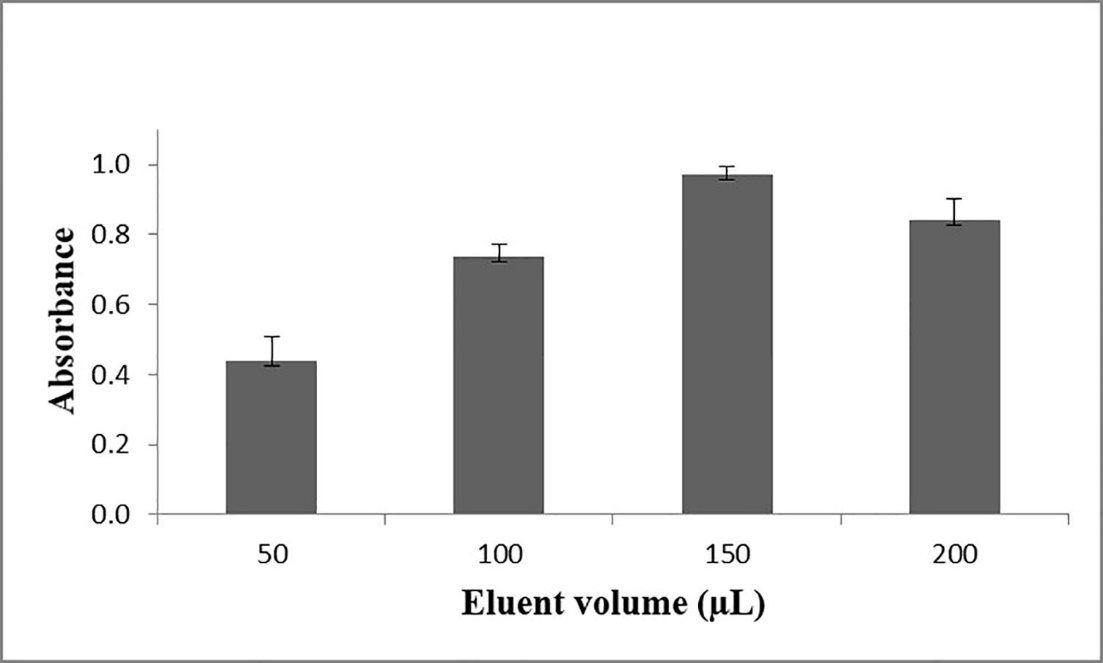
**

**Fig. S6.** Effect of volume of eluent on the extraction efficiency.

**
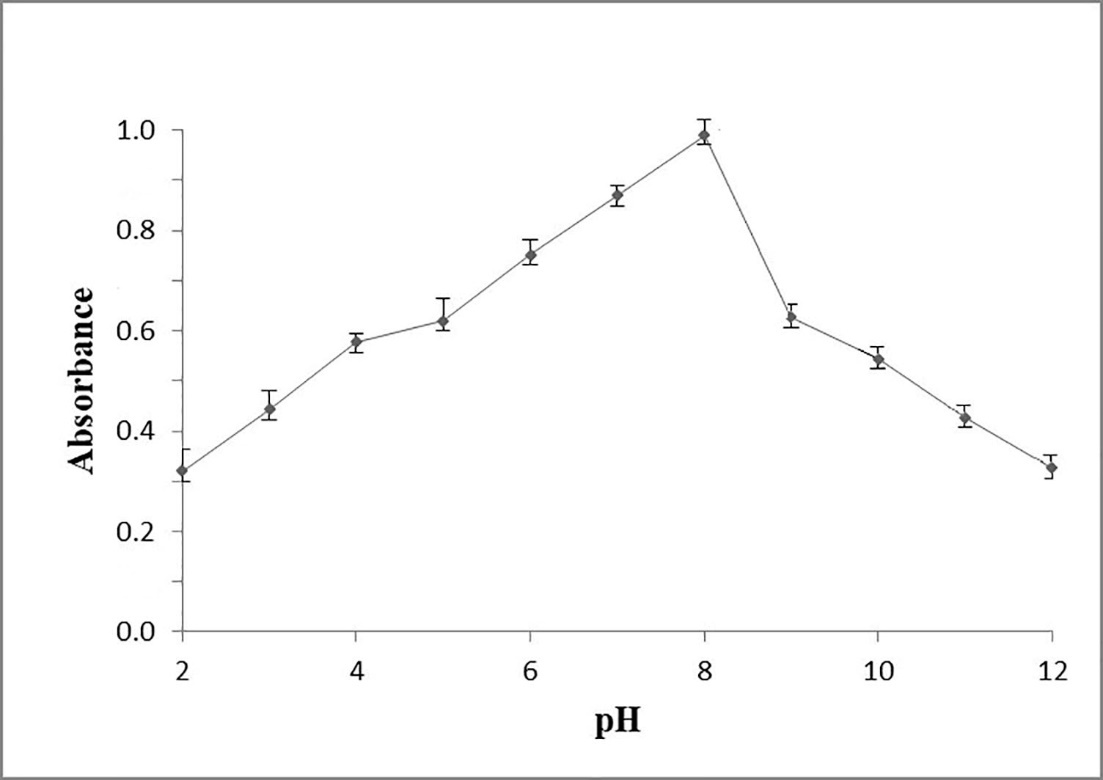
**

**Fig. S7.** Effect of pH on the extraction efficiency.

***
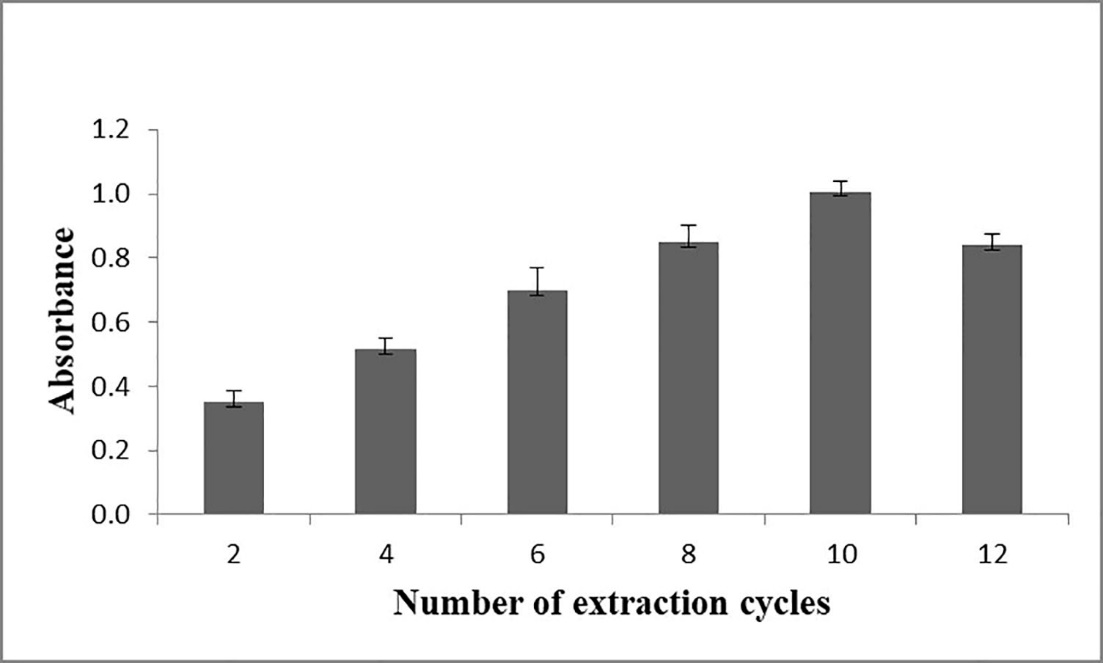
***

**Fig. S8.** Effect of number of extraction cycles on the extraction efficiency.

***
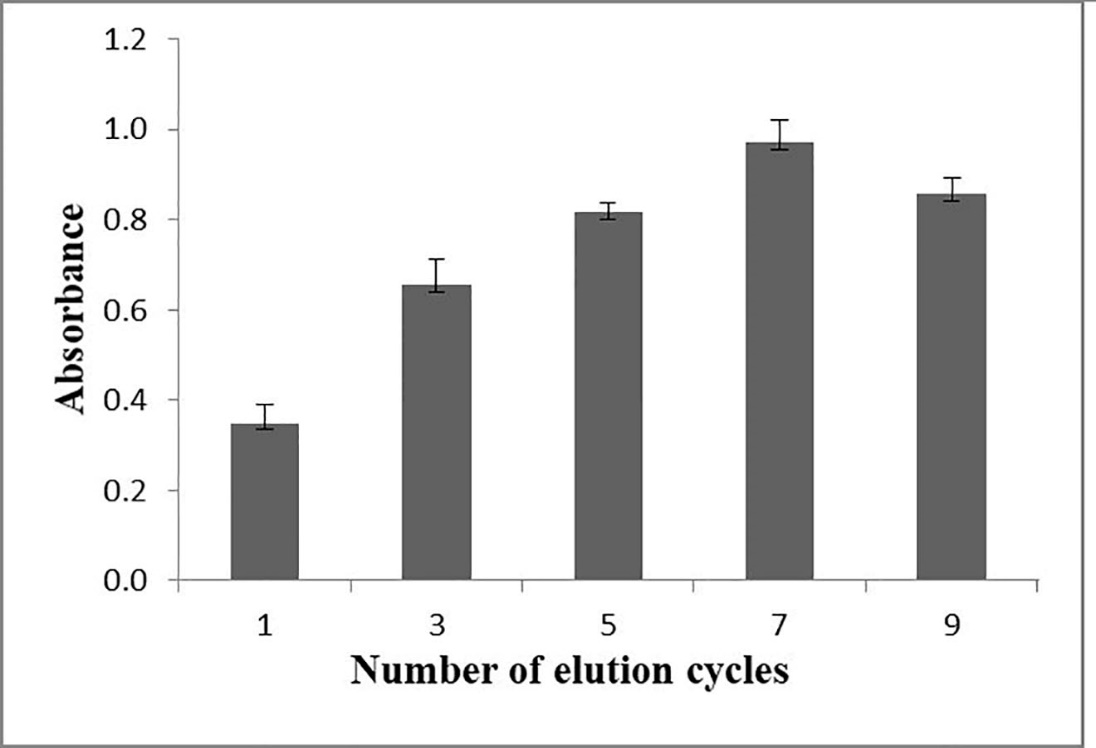
***

**Fig. S9.** Effect of number of elution cycles on the extraction efficiency.

**
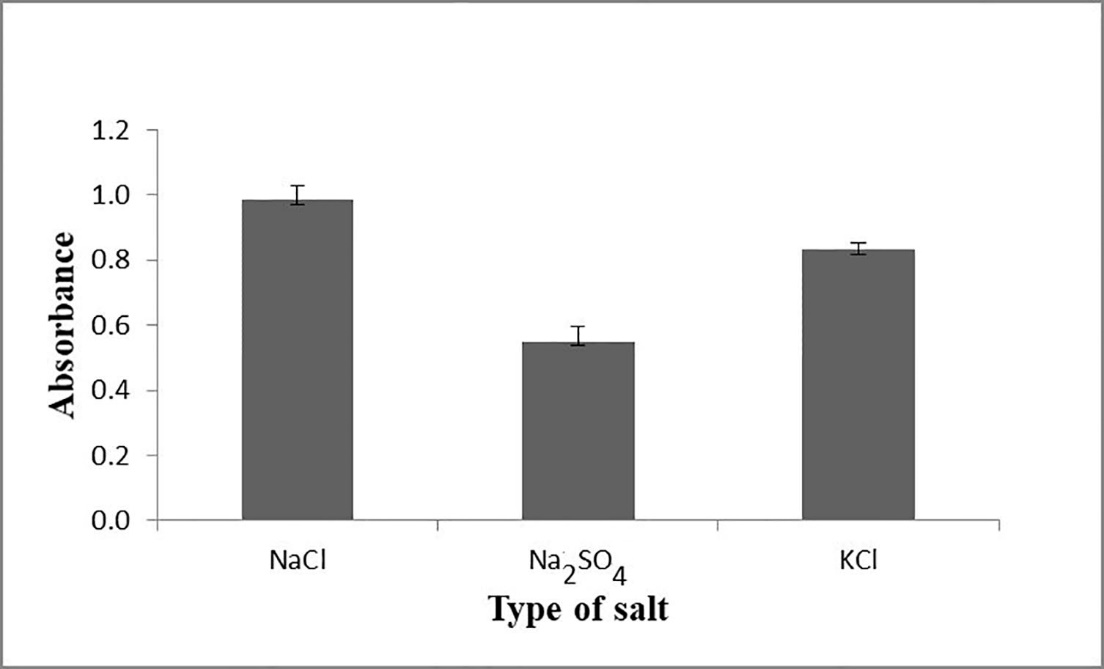
**

**Fig. S10.** Effect of type of salt on the extraction efficiency.

**
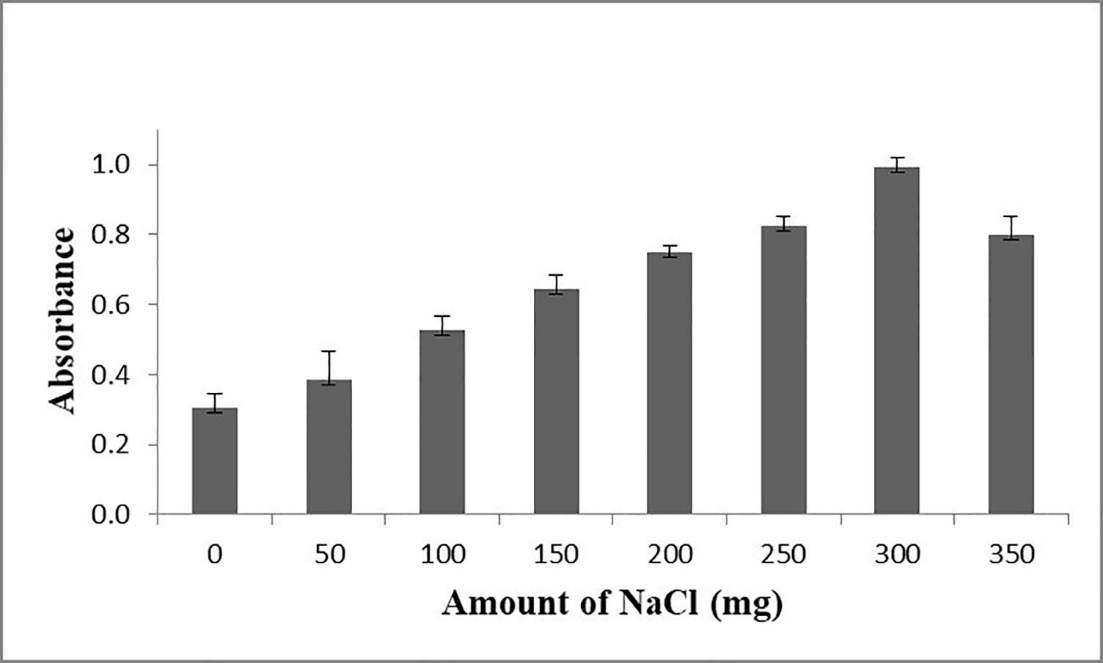
**

**Fig. S11.** Effect of amount of NaCl on the extraction efficiency.

**Table S1.** The obtained performance of DES-CNT-PT-µSPE.

| Feature | Data |
| --- | --- |
| Amount of Sorbent (DES-CNT) | 1.5 mg |
| Type and amount of salt | 300 mg of NaCl |
| Sample volume | 10 mL |
| pH | 8.0 |
| Elution solvent | 150 μL of acetone: acetic acid (1:9, v/v) |
| Extraction cycles | 10 |
| Elution cycles | 7 |
| Limit of detection (LOD) | 0.2 (µg/L) |
| limit of quantification (LOQ) | 0.7 µg/L |
| Linear range | 1.0-1,000.0 µg/L |
| Enrichment factor (EF) | 106 |
| Capacity | 250 µg/g |
| Relative standard deviations (RSDs) | 1.1-4.6% |
| Recoveries | 97.0% to 99.6% |
| Samples | Tap water, seawater and wastewater |
| Time of analysis | 15 min |

**Table S2.** Data details of the LOD and LOQ.

| Data | 0.039, 0.132, 0.167 |
| --- | --- |
| X_average_ | 0.113 |
| ∑ (X_i_-X_average_)^2^ | 0.008433 |
| S_d_= (1/N-1 ∑ (X_i_-X_average_)^2^)^1/2^ | 0.066 |
| LOD= 3S_d_ | 0.198 ~ 0.2 µg/L |
| LOQ= 10S_d_ | 0.66 ~ 0.7 µg/L |

***RSM optimization***

RSM is a multivariate statistical tool and can be applied to predict and study factors affecting a process including extraction. In RSM, response surfaces are plotted as graphical representation to explain the interactive effects of parameters and their consequent effects on signal. Box-Behnken design (BBD) is one of the main factorial designs which is used to assess the quadratic response surface and forming second-order polynomial models in RSM. BBD is a fractional factorial design with 3 levels of fractional factorial design. The present research applied for assessing the impact of process factors on the signal. Optimization involves estimation of coefficients, prediction of signals and checking acceptability of the model. Here, the analytical signal is explained by Eq. 1:

Y = f (X_1_, X_2_, X_3_.………………….X_n_) ± E (1)

Where, Y is the response, f is the absorption function, X_1_…X_n_ are independent parameters, and E is the experimental error.

The polynomial quadratic model can be expressed as Eq. (2):

Y = β_0_ + ∑ β_i_ X_i_ + ∑ β_ii_ X_ii_ + ∑ β_ij_ X_i_ X_j_ + E (2)

Where, Y is the predicted response, β_0_ is the intercept or regression coefficient, β_i_, β_ii_ and β_ij_ are the linear, quadratic and interaction coefficients, X_i_ and X_j_ are coded data of the process parameters and E is the residual/experimental error. For building a quadratic model, runs were performed for three values of each parameter and values were equally spaced. Table S3 shows the real data of the parameters and their corresponding coded values and real experiments.

In this research, BBD was applied to investigating three affecting parameters; i.e., pH (X_1_ or A), volume of eluent (X_2_ or B), number of extraction (X_3_ or C) and elution (X_4_ or D) cycles. Sequential runs were performed to develop the method. Runs were designed for pHs between 7 to 9, volume of eluent between 100 to 200 µL, extraction cycles between 8 to 12 and elution cycles varying between 4 to 8.

The signal function (f) largely relates to the nature of relationship between the signal and the factors (Eq. 3).

Y= (sin (-25.99702 + 5.29671 × A) + (0.015824 × B) + (0.75708 × C) + (0.46076 × D) – (2.24676 × 10^-6^ × A × B) – (5.66623 × 10^-3^ × A × C) + (2.78675 × 10^-3^ × A × D) + (2.93524 × 10^-4^ × B × C) + (1.40314 × 10^-4^ × B × D) + (1.20958 × 10^-4^ × C × D) - (0.32913 × A^2^) – (6.51246 × 10^-5^ × B^2^) – (0.037699 × C^2^) – (0.041666 × D^2^))^2 (3)

The mathematical model produced with the BBD approach was investigated by various statistical parameters i.e., coefficient of determination (R^2^), adjusted R^2^ (R^2^_adj_) and predicted R^2^ (R^2^_pred_). The optimization was applied for setting the highest desirability. As shown in Fig. 4, the calculated factors of maximum signals for 6-MP extraction were achieved at A=7.99, B=150.48 µL, C=10.04 and D=6.06.

Analysis of variance results for the model is given in Tables S4.

The suggested model “F-value” was found to be 19.74, depicting that the model is statistically important. Large F values may occur because of noise and there is only 0.01% of chance for it to be observed. Values of “prob>F” were achieved to be less than 0.0500, explaining that the model terms are important. Model terms A^2^, B^2^, C^2^ and D^2^ are found to be important as well. P values for the model were obtained to be <0.001, proving the effectiveness of the suggested model.

R^2^ values and R^2^_adj_ were found to be 0.9485 and 0.90049. R^2^ values are close to unity which explain better predicting response of the proposed model. The lack-of-fit indicted the failure of the model to show values in regression and the non-important data. Lack-of-fit (>0.05) proves that the model is suitable for the absorptive measurements. The lack-of-fit value of 1.46 shows that the parameter is not suitable due to the pure error. Two dimensional analytical signal surfaces as functions of two agents at the center amount of other parameters are showed in Fig. S12.

**
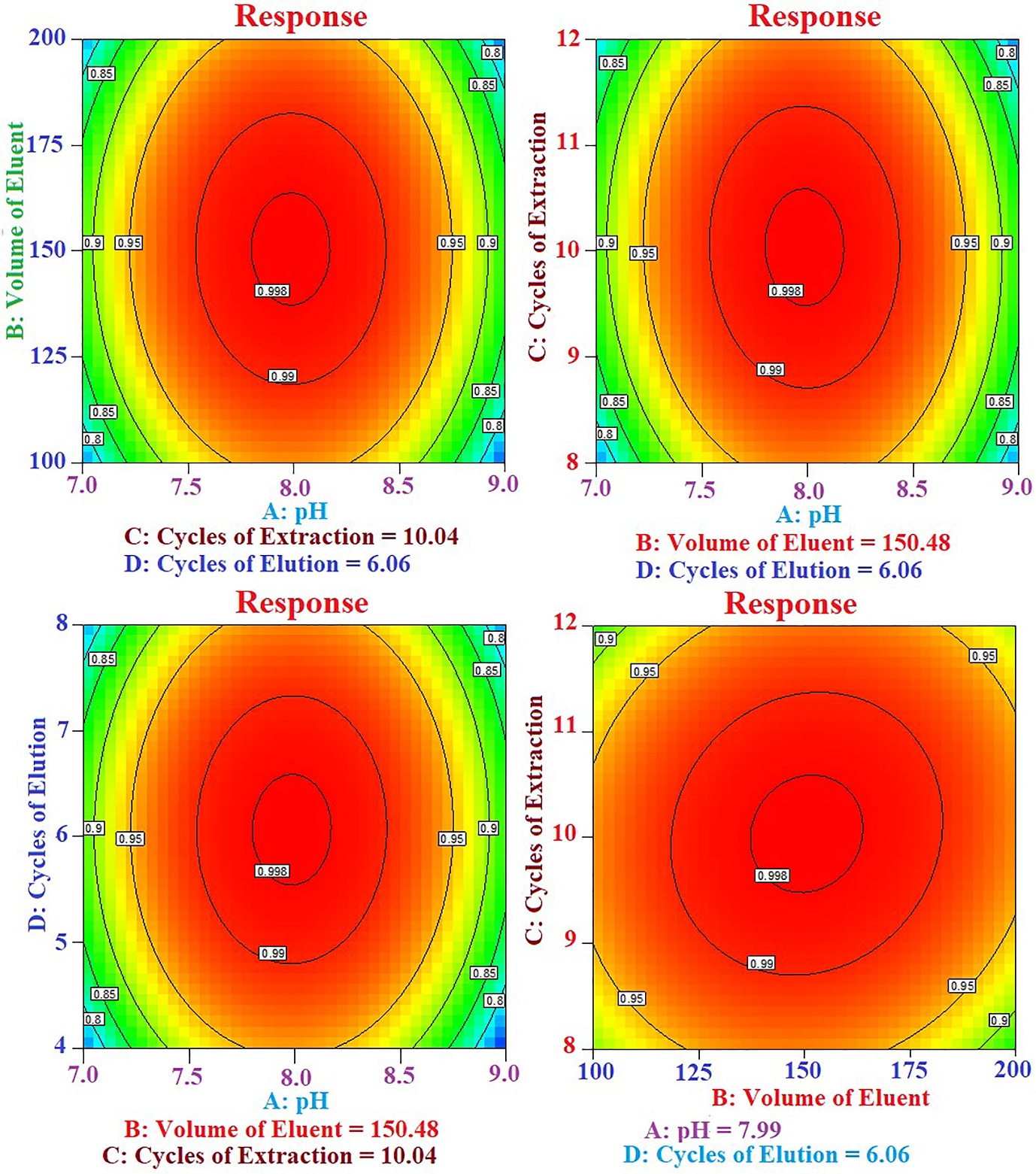
**

**Fig. S12.** Response surface-2D/contours including the effect of the independent variable on the extraction efficiency of 6-MP.

**Table S3.** The design of the actual experiments.

| **No.** | **pH** | **Volume of Eluent** | **Cycles of Extraction** | **Cycles of Elution** | **Response** | **Predicted** | **Error%** |
| --- | --- | --- | --- | --- | --- | --- | --- |
| 1 | 7 | 100 | 10 | 6 | 0.80 | 0.76 | 5.00 |
| 2 | 7 | 150 | 8 | 6 | 0.72 | 0.75 | -4.17 |
| 3 | 7 | 150 | 10 | 4 | 0.74 | 0.75 | -1.35 |
| 4 | 7 | 150 | 10 | 8 | 0.73 | 0.76 | -4.11 |
| 5 | 7 | 150 | 12 | 6 | 0.75 | 0.78 | -4.00 |
| 6 | 7 | 200 | 10 | 6 | 0.81 | 0.76 | 6.17 |
| 7 | 8 | 100 | 8 | 6 | 0.88 | 0.90 | -2.27 |
| 8 | 8 | 100 | 10 | 4 | 0.86 | 0.87 | -1.16 |
| 9 | 8 | 100 | 10 | 8 | 0.86 | 0.87 | -1.16 |
| 10 | 8 | 100 | 12 | 6 | 0.84 | 0.87 | -3.57 |
| 11 | 8 | 150 | 8 | 4 | 0.90 | 0.87 | 3.33 |
| 12 | 8 | 150 | 8 | 8 | 0.92 | 0.89 | 3.26 |
| 13 | 8 | 150 | 10 | 6 | 1.00 | 1.00 | 0.00 |
| 14 | 8 | 150 | 10 | 6 | 1.00 | 1.00 | 0.00 |
| 15 | 8 | 150 | 10 | 6 | 0.99 | 1.00 | -1.01 |
| 16 | 8 | 150 | 10 | 6 | 0.99 | 1.00 | -1.01 |
| 17 | 8 | 150 | 10 | 6 | 1.0 | 1.00 | 0.00 |
| 18 | 8 | 150 | 10 | 6 | 1.0 | 1.00 | 0.00 |
| 19 | 8 | 150 | 12 | 4 | 0.91 | 0.88 | 3.30 |
| 20 | 8 | 150 | 12 | 8 | 0.93 | 0.89 | 4.30 |
| 21 | 8 | 200 | 8 | 6 | 0.85 | 0.86 | -1.18 |
| 22 | 8 | 200 | 10 | 4 | 0.83 | 0.86 | -3.61 |
| 23 | 8 | 200 | 10 | 8 | 0.87 | 0.89 | -2.30 |
| 24 | 8 | 200 | 12 | 6 | 0.89 | 0.91 | -2.25 |
| 25 | 9 | 100 | 10 | 6 | 0.78 | 0.74 | 5.13 |
| 26 | 9 | 150 | 8 | 6 | 0.74 | 0.76 | -2.70 |
| 27 | 9 | 150 | 10 | 4 | 0.70 | 0.72 | -2.86 |
| 28 | 9 | 150 | 10 | 8 | 0.71 | 0.75 | -5.63 |
| 29 | 9 | 150 | 12 | 6 | 0.73 | 0.75 | -2.74 |
| 30 | 9 | 200 | 10 | 6 | 0.79 | 0.74 | 6.33 |

**Table S4.** Analysis of variance of BBD model for 6-MP extraction.

| **Source** | **Sum of**  **Squares** | **df** | **Mean**  **Square** | **F**  **Value** | **p-value**  **Prob > F** | **Significant/**  **nonsignificant** |
| --- | --- | --- | --- | --- | --- | --- |
| Model | 0.966451 | 14 | 0.069032 | 19.74483 | < 0.0001 | significant |
| A-pH | 0.001135 | 1 | 0.001135 | 0.324512 | 0.5773 |  |
| B-Volume of Eluent | 6.27E-05 | 1 | 6.27E-05 | 0.017932 | 0.8953 |  |
| C-Cycles of Extraction | 0.000306 | 1 | 0.000306 | 0.087475 | 0.7715 |  |
| D-Cycles of Elution | 0.001359 | 1 | 0.001359 | 0.388589 | 0.5424 |  |
| AB | 5.05E-08 | 1 | 5.05E-08 | 1.44E-05 | 0.9970 |  |
| AC | 0.000514 | 1 | 0.000514 | 0.14693 | 0.7069 |  |
| AD | 0.000124 | 1 | 0.000124 | 0.03554 | 0.8530 |  |
| BC | 0.003446 | 1 | 0.003446 | 0.985706 | 0.3365 |  |
| BD | 0.000788 | 1 | 0.000788 | 0.225251 | 0.6419 |  |
| CD | 9.36E-07 | 1 | 9.36E-07 | 0.000268 | 0.9872 |  |
| A^2 | 0.742833 | 1 | 0.742833 | 212.4675 | < 0.0001 |  |
| B^2 | 0.181766 | 1 | 0.181766 | 51.98943 | < 0.0001 |  |
| C^2 | 0.155927 | 1 | 0.155927 | 44.5987 | < 0.0001 |  |
| D^2 | 0.190468 | 1 | 0.190468 | 54.47835 | < 0.0001 |  |
| Residual | 0.052443 | 15 | 0.003496 |  |  |  |
| Lack of Fit | 0.039065 | 10 | 0.003907 | 1.460054 | 0.3543 | not significant |
| Pure Error | 0.013378 | 5 | 0.002676 |  |  |  |
| Cor Total | 1.018894 | 29 |  |  |  |  |

1. Corresponding author. Tel: +98(54)33446413; Fax:+98(54)33431067; E-mail: kaykhaii@chem.usb.ac.ir

   ^1^Department of Marine Chemistry, Faculty of Marine Science, Chabahar Maritime University, Chabahar, Iran

   ^2^Department of Mechanical Engineering, Faculty of Marine Engineering, Chabahar Maritime University, Chabahar, Iran

   ^3^Department of Chemistry, Faculty of Sciences, University of Sistan and Baluchestan, Zahedan 98135-674, Iran [↑](#footnote-ref-1)
